# An Atomic Force Microscopy Study on the Effect of β-Galactosidase, α-l-Rhamnosidase and α-l-Arabinofuranosidase on the Structure of Pectin Extracted from Apple Fruit Using Sodium Carbonate

**DOI:** 10.3390/ijms21114064

**Published:** 2020-06-05

**Authors:** Piotr Mariusz Pieczywek, Justyna Cybulska, Artur Zdunek

**Affiliations:** Institute of Agrophysics, Polish Academy of Sciences, Doświadczalna 4, 20–270 Lublin, Poland; j.cybulska@ipan.lublin.pl (J.C.); a.zdunek@ipan.lublin.pl (A.Z.)

**Keywords:** pectin, apple fruit, atomic force microscopy, enzymatic degradation

## Abstract

The enzyme driven changes in plant cell wall structure during fruit ripening result in debranching, depolymerization and solubilization of pectin polysaccharides, which has an effect in terms of the postharvest quality losses in fruit. Atomic force microscopy (AFM) has revealed that diluted alkali soluble pectins (DASP) from fruit and vegetables have an interesting tendency to self-assemble into regular structures. However, the mechanism is not yet fully understood. The current study is aimed at investigating the role of neutral sugars, namely galactose, rhamnose and arabinose in the formation of the branched structure of DASP. β-galactosidase, α-l-rhamnosidase and α-l-arabinofuranosidase enzymes were used for the treatment of DASP extracted from Golden Delicious apple flesh (*Malus domestica* cv. Golden Delicious). The effects of the selective degradation of pectic polysaccharides after 15, 30, 60, 90 and 120 min of incubation were observed using AFM. The α-l-rhamnosidase enzyme activity on pectin extracted with Na_2_CO_3_ did not cause any visible or measurable degradation of the molecular structure. The moderate effects of β-galactosidase enzymatic treatment suggested the possible role of galactose in the branching of DASP molecules deposited on mica. Data obtained for α-l-arabinofuranosidase indicated the crucial role of arabinose in the formation and preservation of the highly branched structure of the DASP fraction.

## 1. Introduction

The plant cell wall of flesh fruit tissue is a heterogeneous matrix of interacting and cross-linked polysaccharides, which provides structural integrity for the whole fruit. Being a Type I primary cell wall, it is a network of xyloglucan-cellulose microfibrils, embedded and closely linked with an amorphous matrix of pectin polysaccharides [1,2,3,4]. Although the xyloglucan-cellulose network is considered to be the basic load-bearing component of the plant cell wall, the enzyme-induced modifications of the pectin matrix are now gaining more recognition as the major cause of postharvest quality losses in apples [5,6]. The main unit which comprises approximately 70% of all pectins, is D-galacturonic acid (GalA). Other pectin components include a variety of neutral sugars such as arabinose, galactose, fucose, rhamnose, etc. At a higher level of structural hierarchy, the pectic polysaccharides of the plant cell wall may be divided into four different domains, depending on their galacturonic acid content: homogalacturonan (HG), rhamnogalacturonan I (RGI), rhamnogalacturonan II (RGII) and xylogalacturonan [7]. The enzyme-driven changes in plant cell wall structure during fruit ripening result in the deesterification, debranching, depolymerization and solubilization of pectin polysaccharides [6,8]. Breakdown of the cell wall and further loss of middle lamella integrity leads to substantial changes in fruit texture and finally to quality losses. Several pectin-degrading enzymes such as polygalacturonase, pectin methyl esterase, β-galactosidase, pectate lyase and α-arabinofuranosidase have been linked to fruit softening [9,10]. It is commonly assumed that the first pectin modification to occur is the cleavage of the neutral sugars of the side chain from the polymer backbone through the action of β-galactosidase and α-l-arabinofuranosidase. It was assumed that this increases cell wall porosity, thereby facilitating the action of pectin methylesterase [6,11]. Studies have shown that the pectin fraction extracted with chelating or alkaline reagents, the ionic and covalently bound pectin respectively, are the cell wall pectin fractions that undergo the most substantial structural changes during fruit softening [11,12,13].

The diluted alkali-soluble fraction of pectin (DASP) extracted from apples, that represents the covalently linked molecules in the cell wall, is mainly composed of galacturonic acid, arabinose and the much less abundant galactose and rhamnose. A previous atomic force microscopy (AFM) study has shown that the DASPs extracted from fresh fruits and vegetables such as carrot, apple or pear, form a regular interlinked network when they dry out on mica [14,15,16]. Thus, the DASP fraction, quantitatively dominating in cell walls of fruit tissue, probably has the most crucial role in maintaining cell wall spatial architecture due to its self-assembling properties [16,17]. Moreover, the AFM studies have shown that the degradation of the DASP network is correlated with the duration of fruit storage [15]. Nanoindentation experiments have also demonstrated a negative correlation between cell wall stiffness and the content of galacturonic acid in the pectin fraction which is covalently linked in pear cell walls [17]. However, the exact role of the individual compounds of the DASP fraction in the mechanism of molecular branching and supramolecular assembly is not fully understood yet. In most studies concerning the effects of enzyme-induced changes on pectin structure, chemical methods and enzyme activity assays are used [18,19,20,21]. Controlled mild acid hydrolysis of sodium carbonate extracted pectin resulted in degradation of the RGI complexes observed with AFM but did not influence significantly on the individual HG chains and HG domains in the complexes [22]. In vitro enzymolysis of pectin extracted from citrus peel performed using pectinase resulted in shortening and debranching of long and folded pectin chains [23]. However, the action of specific pectin-degrading enzymes on the nanostructure of DASP fraction has not been previously described.

In this paper we propose to supplement AFM imaging with objective and quantitative image analysis techniques to study the effects of selective enzymatic degradation of DASP. The role of the three neutral sugars: galactose, rhamnose and arabinose in the formation of the branched structure of DASP was investigated using β-galactosidase (GAL), α-l-rhamnosidase (RHA) and α-l-arabinofuranosidase (ABF) enzymes. The effects of selective degradation after different times of incubation were observed using AFM.

## 2. Results

**Figure 1:**
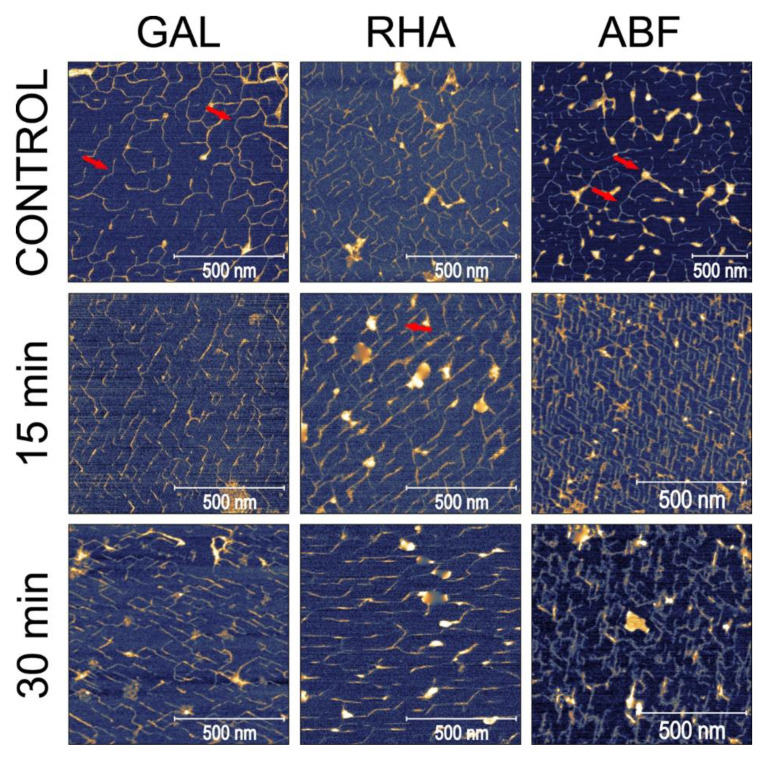
Sample images of DASP treated with GAL, RHA and ABF with respect to the duration of enzyme treatment (arrows indicate local bend points and aggregates).

**Figure 2:**
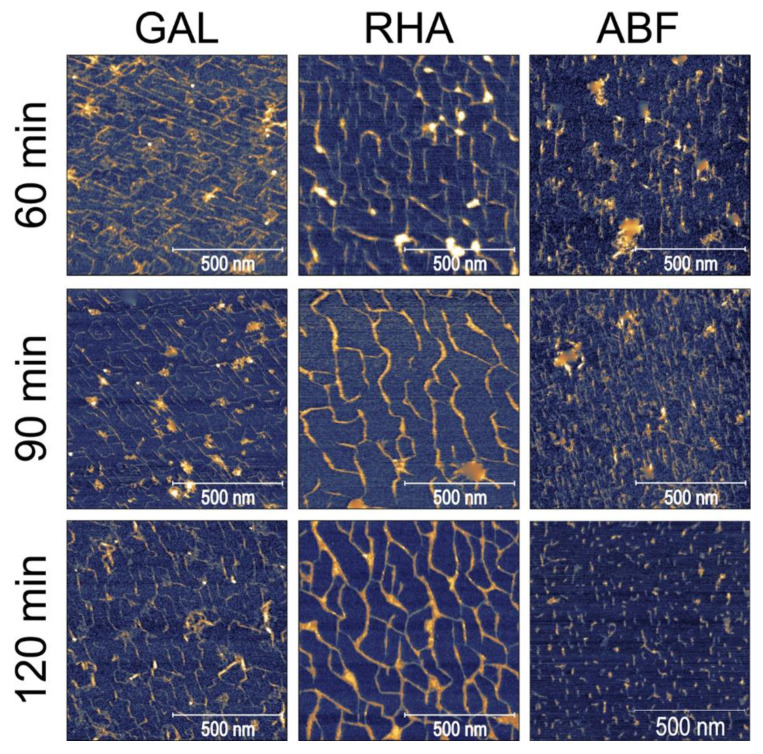
Sample images of DASP treated with GAL, RHA and ABF with respect to the duration of enzyme treatment.

**Figure 3:**
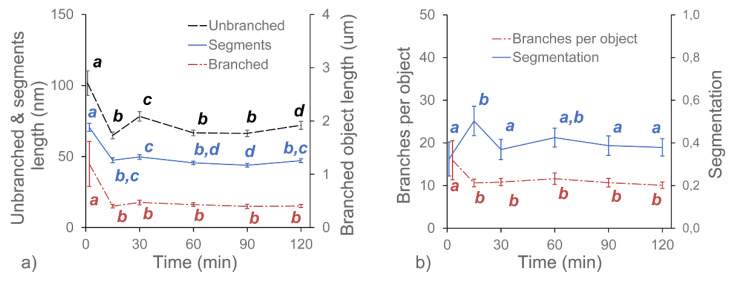
Changes in the average values of the structural parameters of DASP treated with GAL with respect to the duration of enzyme treatment. Different letters indicate significant differences (according to ANOVA with *p* = 0.05). Bars indicate 95% confidence intervals for mean values.

**Figure 4:**
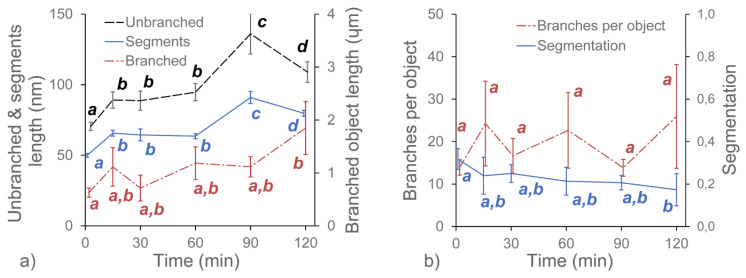
Changes in the average values of the structural parameters of DASP treated with RHA with respect to the duration of enzyme treatment. Different letters indicate significant differences (according to ANOVA with *p* = 0.05). Bars indicate 95% confidence intervals for mean values.

**Figure 5:**
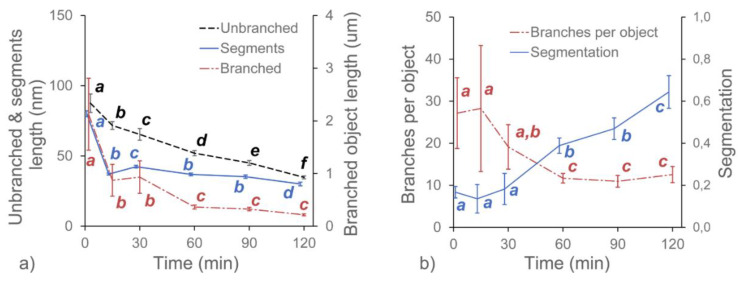
Changes in the average values of the structural parameters of DASP treated with ABF with respect to the duration of enzyme treatment. Different letters indicate significant differences (according to ANOVA with *p* = 0.05). Bars indicate 95% confidence intervals for mean values.

**Figure 6:**
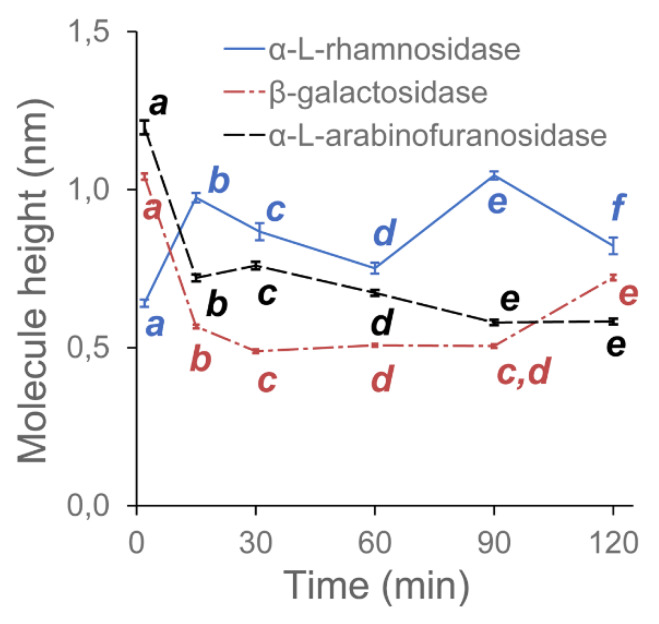
Changes in the diameter of DASP molecules with respect to the duration of enzyme treatment. Different letters indicate significant differences (according to ANOVA with p = 0.05). Bars indicate 95% confidence intervals for mean values. The diameter was defined as the maximum height value measured at the central line of the molecule (skeleton).

In this experiment, we investigated the effects of three pectolytic enzymes on the macromolecular structure of the diluted alkali-soluble fraction of pectin extracted from apple fruit. GAL, RHA and ABF were chosen to reduce the lengths of the neutral sugar molecular chains and modify the structure of DASP. The effect of the selective degradation of pectic polysaccharides after 15, 30, 60, 90 and 120 min of incubation were observed using AFM. Each of the enzymes required different buffers at various pH levels. Since pH is known to have an impact on the structure of pectin [24], three control samples of DASP in their corresponding buffers were prepared. Sample images of the control samples and DASP after different enzyme incubation times are shown in Figure 1 and Figure 2. Figure 3, Figure 4, Figure 5 and Figure 6 show the results of an AFM image analysis for GAL, RHA and ABF treated samples, respectively.

The images of the untreated samples show structural features characteristic of apple DASP, which were also observed in previous studies [16]. The DASP deposited on mica-formed branched structures consisting of linear sections interrupted by local bend points or branches, with aggregates concentrated around junction points of linear molecules (Figure 1 control for GAL, RHA and ABF). The average lengths of the unbranched structures ranged from 70.4 through to 87.5 and up to 104.0 nm (Figure 3a, Figure 4a and Figure 5a, respectively). These values corresponded very well with previous studies where the reported total lengths of the unbranched structures were 87.4 nm on average. The control samples showed differences with respect to the number of branches per object, the size of the branched objects and the level of segmentation of the objects, with the highest levels of branching and the largest branched structures being reported for the ABF-buffer and the less branched and smaller structured ones for the GAL- and RHA-buffers. These differences have been mainly attributed to the differences in the pH levels of the applied buffers.

GAL is an enzyme which hydrolyzes the β-glycosidic bond formed between a galactose and its organic moiety [25]. It may also hydrolyze some α-l-arabinosides and β-d-fucosides but with much lower efficiency [26]. In the generally accepted view of pectin structure, galactose is a part of the side chains of the rhamnogalacturonan-I backbone, which are commonly known as “hairy regions” of pectin. Based on the reported relative amounts of galactose and rhamnose in DASP extracted from apples (4.17% and 3.75%, respectively according to [16]), the impact of GAL was expected to be low. However, a significant decrease in the average lengths of all three groups of objects was observed after 15 min of the incubation of DASP in GAL solution (Figure 3a).

The initial decrease in molecular lengths was not followed by a further decrease during the next two hours of incubation. The lengths of the objects obtained after 15 min of incubation remained constant up to the end of the experiment (with exception of increased lengths of unbranched objects after 30 min). A similar trend was observed for the number of branches per object and the molecular diameters (Figure 6), which remained constant after a small initial decrease (Figure 3b). In contrast, trend in changes in the segmentation of the DASP molecules affected by β-galactosidase treatment was not statistically significant, although values of this parameter were slightly higher for enzymatically treated samples than for the untreated (Figure 3b).

Compared to the GAL samples, the RHA-treated ones showed opposite trends in changes to molecule lengths. The lengths of the unbranched and branched objects (Figure 4a) as well as the number of segments of branched objects showed a gradual and statistically significant increase in values during incubation in RHA solution. The corresponding average number of branches per object showed a relatively high variability but the changes were not significant (Figure 4b). Data also showed that the segmentation of molecular objects from AFM images decreased with the incubation time. The geometrical parameters of the molecular structures indicated that during 120 min of incubation, a process of polysaccharide aggregation occurred. The results of the measurements were in agreement with visual data from Figure 1 and Figure 2, which showed an increase in the thickness of the molecules, with a corresponding increase in separation distance between the chains and the distance between branching points (lengths of segments). An increase in the thickness of the molecules was also confirmed by measuring the molecular diameters (Figure 6), which exceeded the diameter of a single molecular chain of homogalacturonan.

Among the three tested enzymes, ABF showed the highest effect on the structure of covalently linked DASP molecules. The AFM images show the gradual degradation of the DASP structure from a highly branched network (Figure 1, control) to almost completely depolymerized and debranched individual molecules (Figure 2, 120 min). Visual observations were supported by the results of the calculation of the geometrical parameters of DASP molecules. The lengths of the individual unbranched objects, the number of segments of the branched objects and the total lengths of the branched structures showed a significant decrease after the application of ABF (Figure 5a). A gradual decrease in molecular lengths and diameters (Figure 6) was observed during the whole incubation period. Along with the length of the molecules, a significant decrease in the average number of branches per object was observed for ABF treated samples (Figure 5b). The depolymerization and debranching of DASP was also confirmed by an increase in the number of unbranched structures, indicated by an increase in the values of segmentation (Figure 5b). The data obtained indicates the crucial role of arabinose in the formation and preservation of the highly branched structure of the DASP fraction.

## 3. Discussion

GAL, RHA and ABF showed different effects of the selective degradation of DASP pectic polysaccharides. The observed effects of GAL enzymatic treatment suggested the possible role of galactose in the branching of DASP molecules deposited on mica. Based on a chemical analysis combined with molecular dynamics modeling it was shown that, in the DASP fraction, rhamnose is present in the form of single rhamnose interspersions or short RGI sections between two homogalacturonan chains [16,27]. When considering galactose as a side chain attached to rhamnose units, the currently observed branching mechanism could be explained by bridging two rhamnose units from separate molecular chains with galactose. Degradation of such connections by GAL could result in a decrease in the size of objects and a decrease in the number of branches per object, which were both observed in this study. The amount of GAL added to the DASP solution was calculated on the basis of galactose content and the specific activity of the enzyme. However, gradual changes in the structural parameters have not been observed. The enzyme acted immediately in the first 15 min of incubation, causing the initial drop in molecule lengths. This could be explained by the selective activity of the GAL used which catalyzes the hydrolysis of terminal non-reducing β-d-galactose residues in β-d-galactosides. It means that a significant amount of galactose could occur in the form of arabinogalactan subunits, which are not susceptible to β-galactosidase activity [28]. The RHA enzyme is responsible for the hydrolysis of terminal non-reducing α-l-rhamnose residues in α-l-rhamnosides. Therefore, the lack of visible and measurable enzymatic degradation supported previous suppositions that rhamnose forms single residue interspersions or short RGI sections in homogalacturonan chains from diluted alkali-soluble fraction of apple pectin, which are not accessible for RHA [16]. This hypothesis was also supported by the presence of characteristic bend points of molecular chains in all tested samples (Figure 1 and Figure 2). Moreover, the results confirm that the alternative proposed structures, in which HG is a long side chain of RGI were not present in the DASP fraction [29].

The high impact of ABF on the structure of DASP could be demonstrated by the relatively high content of arabinose in this fraction of pectin. Previous studies have shown that arabinose is a highly abundant component of the DASP extracted from Golden Delicious apples. It accounted for up to 29% of the total chemical composition of the DASP fraction [16]. The most abundant compound of DASP, galacturonic acid, is present mainly in the form of HG polymeric chains. Therefore, it may be assumed that arabinose is able to link the neighboring HG molecules to eventually form a network; this may be observed on the mica surface. The exact molecular mechanism of linking is unknown. However, the results suggest that arabinose may play a crucial role in plant tissue mechanics and plant tissue softening by maintaining the branched structure of DASP homogalacturonan molecules. For example, the increased activity of the ABF enzyme may decrease the connectivity of covalently linked pectin molecules and therefore reduce the resistance of the cell wall to mechanical load. This theory is in line with multiple studies indicating that the presence or deficiencies of arabinose in plant cell walls significantly alters their mechanical response to induced loads. Kozlova et al. [30] showed that specific α-l-arabinofuranosidases are involved in the realization of the elongation growth process in cells with type II cell walls (possess by commelinoid monocots, similar to type I wall contain cellulose microfibrils, but interlocked mostly by glucuronoarabinoxylans instead of xyloglucans [1]). The authors showed that the measured enzymatic activity and free arabinose content significantly increases during maize (*Zea mays* L.) root elongation. Studies with molecular probes directed to the structural elements of pectic polymers indicated that arabinan structure/processing is probably involved in the local control of cell wall mechanical properties [31]. The reduction in the number of pectic arabinan side chains was also identified as a possible mechanism for pollen cell wall failure [32]. Other studies provided biochemical evidence for arabinan functioning in relation to the cell adhesion process [10,33,34]. Studies concerning apple softening have associated this process with the loss of side chain galactose and arabinose from the RGI side chain of the cell wall pectin and the increased activities of GAL and ABF [8,10,35]. Gwanpua et al. [5,8] suggested that the loss of arabinose from the side chains of RGI, and pectin depolymerization could be events associated with late ripening. However, the relatively low amount of rhamnose reported for DASP extracted from Golden Delicious apple flesh calls into question the role of RGI side chain mechanism of associations. It is not clear if such a small amount of RGI and therefore arabinose associated with the RGI backbone could explain such a dramatic degradation as has been observed in this study. Other possible types of arabinose-homogalacturonan polysaccharide associations may be responsible for DASP cross-linking. A proper chemical linkage analysis is necessary in order to obtain a comprehensive quantitative view of branch points and polysaccharide.

## 4. Materials and Methods

### 4.1. Apple Fruit

Apple fruit (*M. domestica* cv. Golden Delicious) were used in this study as a source of pectin. The sample material was purchased in the local market. Fruit were peeled and the apple flesh (parenchyma tissue) was processed into pulp.

### 4.2. Cell Wall and DASP Extraction

Randomly selected fruit were used in the preparation of alcohol-insoluble residue (AIR) according to the method described by [36] with slight modifications. The apples were peeled, homogenized and mixed with ∼70% ethanol for 30 min. Next, the mixture was filtered and stirred again with ethanol until a negative result was achieved for the phenol-sulfuric acid assay for total sugar [37]. Then, the suspension was washed with 96% ethanol and acetone and finally dried at 40 °C.

The sequential extraction of the pectin fractions was conducted using a method reported by [38] with slight modifications [39]. The AIR sample was mixed with deionized water for 24 h and then filtered through a nylon filter. Then, 0.05 M trans-1,2-diaminocyclohexane-N,N,N’,N’-tetraacetic acid (CDTA, pH 6.5) was then added to the sediment and this suspension was mixed for 24 h. After filtration, the residue was stirred with 0.05 M Na_2_CO_3_ + 0.02 M NaBH_4_ for 24 h. The sample was filtered and the filtrate was collected as the diluted alkali-soluble pectin (DASP fraction). After collecting 100 μL of DASP solution for AFM imaging, the rest of the DASP sample was lyophilized and used for analytical studies.

### 4.3. Enzymatic Treatment of DASP

The following commercially available enzymes were used in the experiment: β-galactosidase (GAL, E.C. 3.2.1.23) with a specific activity of 170 U/mg (40 °C, pH 4.5 on *p*-nitrophenyl-β-d-galactoside), isolated from *Aspergillus niger*, α-l-rhamnosidase (RHA, E.C. 3.2.1.40) with a specific activity of 190 U/mg (50 °C, pH 6.5 on *p*-nitrophenyl-α-l-rhamnoside), isolated from a prokaryote and α-l-arabinofuranosidase (ABF, E.C. 3.2.1.55) with a specific activity of 32 U/mg (40 °C, pH 5.5 on *p*-nitrophenyl-α-l-arabinofuranoside), isolated from *Aspergillus niger*. All of the enzymes were purchased from Megazyme International Ireland (Bray Business Park, Bray, Co. Wicklow, Ireland). Enzymatic hydrolysis was performed in glass vials containing approximately 0.5 mg of DASP dissolved in 10 mL of an optimal buffer (0.005% *w/v*). The amounts of the enzymes added to the DASP solutions were calculated on the basis of their specific activities and the chemical composition of the DASP fraction which contained about 202.24 mg of arabinose, 32.23 mg of rhamnose and 31.72 mg of galactose in 1 g DASP [16]. For each enzymatic treatment, six samples were prepared: control, without incubation, those with previously selected incubation times: 15, 30, 60, 90 and 120 min. For the GAL treatment 100 mM of acetate buffer (pH 4.5) was used. Then, 3.8 µL of GAL was added to each test tube and hydrolysis was performed at 40 °C in a water bath. For RHA treatment, 100 mM sodium phosphate buffer (pH 6.5) was used, with the addition of 2 µL of RHA and incubation at 50 °C. For ABF treatment, 100 mM sodium acetate buffer (pH 4.0) was used, with the addition of 10.7 µL of ABF and incubation at 50 °C. After their specific incubation time was complete the samples were vortexed and immediately distributed on mica sheets using a spin coater.

### 4.4. AFM Imaging and Image Analysis

**Figure 7:**
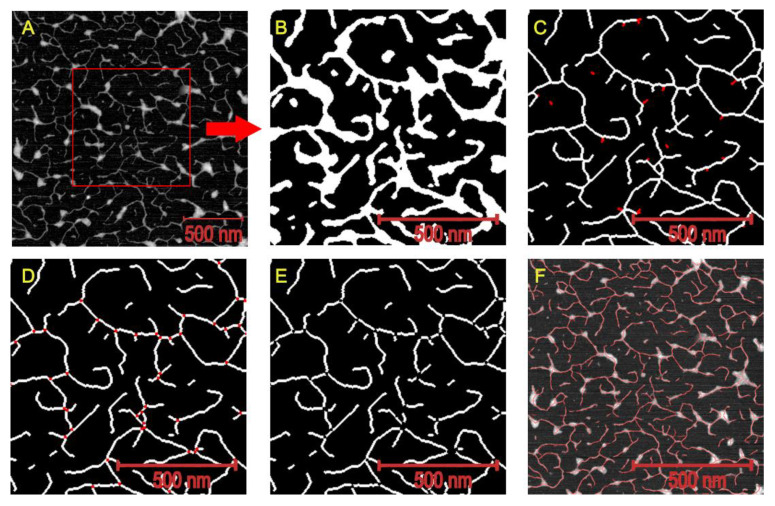
AFM image of DASP and subsequent stages of image analysis: (**A**) raw AFM image, (**B**) results binary thresholding and median filtering, (**C**) skeletonization, short side-branches and short individual molecules removal (removed objects indicated using red color), (**D**) branch points identification (red color), (**E**) object debranching, (**F**) raw image with imposed molecule skeletons.

The diluted alkali soluble pectins (DASP) were mixed with the enzyme and buffer solutions and then 15 μL of this solution was deposited on the mica surface using a spin coater (POLOS SPIN150i NPP, SPS-Europe B.V., Putten, the Netherlands). The samples were dried in a desiccator at 22 °C overnight prior to AFM observation. The images were captured using a Multimode 8 with a Nanoscope V controller (Bruker, Billerica, MA, USA), with SCANASYST-AIR HR cantilever (Bruker, Billerica, MA, USA). For each image a rectangular area of 1 μm^2^ (1 × 1 μm) or 4 μm^2^ (2 × 2 μm) was scanned with a resolution equal to 512 × 512 points, depending on the size of the visible structures. All images were initially pre-processed using Gwyddion 2.52 [40]. The extraction of the skeletal structures of molecules (Figure 7) and the calculations of geometrical parameters were performed using the Matlab R2011a application (MathWorks, Natick, MA, USA). All AFM images were initially filtered using a 5 × 5 Gaussian filter with a standard deviation equal to 1.0. Next, simple segmentation by binary thresholding of molecules heights was carried out in order to separate the pectic molecules from the background of the image. The level of height threshold was equal to 0.1 nm. The resulting binary images were filtered using 5 × 5 median filter in order to smooth the boundaries of all identified objects (Figure 7B). Due to presence of segmentation artefacts the objects from binary images were also filtered according to their areas. All objects with area less than 100 nm^2^ were excluded from the further analysis. Cleaned images were skeletonized (objects were thinned to lines) by means of the standard binary morphological operators. For objects with “jagged” edges the skeletonization procedure may produce additional artefacts as short side-branches of skeletons. Therefore, a corrective procedure was implemented, which identified and removed skeleton side-branches with length less than 5 pixels (Figure 7C). The same threshold level was used to remove all unconnected individual skeleton fibres. Finally, branch points of the skeletons were identified and removed in order to obtain thin, non-connected lines representing the individual fibers (Figure 7 D,E). Binary images of detached segments were used to determine the sampling points of molecule diameters on the original topological images. Diameter sampling points were determined every 10 pixels starting from a point 5 pixels away from one end of the fibre. The diameter of the molecule was defined as the maximum height value within a 3 × 3 pixel window around each sampling point.

All of the objects from the AFM images were classified into two categories—branched and unbranched objects (Figure 7D). The lengths of the branched and unbranched molecules were calculated individually. Additionally, the lengths of the linear segments of the branched objects were calculated (lengths of individual molecules contributing to branched objects). The length of the linear segment of the branched object was defined as the length of the linear section starting from the branch point to the nearest bend, branch or molecule end. The structure of the branched objects was described by the average number of branches per object. The classification of objects from AFM images was defined as the ratio of the number of unbranched objects to the total number of linear segments of branched objects and unbranched objects together. At least 15 images were taken for each enzymatic treatment and incubation time. For height calculations, 4000 sampling points were chosen for each enzyme/incubation time. In the case of the molecule length calculations the number of analyzed objects varied depending on enzymatic treatment and incubation time. For the GAL treatment on average 2406 (from 1715 to 3175), 276 (from 190 to 354) and 2181 (from 1903 to 2907) objects were analyzed for segments lengths, branched objects lengths and unbranched object lengths, respectively. For the RHA treatment on average 2401 (from 566 to 3701), 154 (from 53 to 268) and 789 (from 354 to 1600) objects were analyzed for segments lengths, branched objects lengths and unbranched object lengths, respectively. For the ABF treatment on average 3536 (from 1245 to 6545), 236 (from 149 to 349) and 1772 (from 747 to 3471) objects were analyzed for segments lengths, branched objects lengths and unbranched object lengths, respectively.

## 5. Conclusions

In this study the role of neutral sugars, namely galactose, rhamnose and arabinose in the formation of the DASP branched structure was investigated by means of selective enzymatic degradation. The RHA enzyme activity on pectin extracted with Na_2_CO_3_ did not cause any visible or measurable degradation in the molecular structure of pectin. With regard to the reported amount of rhamnose in DASP, this observation supported the existence of short sections of RGI or single interspersions of rhamnose between the two linear sections of the homogalacturonan chains. Such single rhamnose inclusions were previously identified as characteristic bend points of the linear sections of pectic molecules. Structures of this kind were also observed in this study in the case of all of the tested enzymes and incubation periods. The moderate effects of GAL enzymatic treatment suggested the possible role of galactose in the branching of DASP molecules deposited on mica. Significant structural changes were reported after 15 min of treatment with no further enzymatic degradation for longer incubation times. The initial rapid activity of the enzyme which was followed by a lack of further changes in DASP structure may be explained by the high contribution of galactose in arabinogalactan subunits, which are not liable to GAL driven hydrolysis. The data obtained for the ABF treated samples indicated the crucial role of arabinose in the formation and preservation of the highly branched structure of the DASP fraction. Among the three enzymes tested, ABF showed the greatest effect on the structure of the covalently linked DASP molecules, causing the degradation of the DASP structure from a highly branched network to almost completely depolymerized and debranched individual molecules. The exact molecular mechanism of linking is unknown, but the results suggest that arabinose may play a crucial role in plant tissue mechanics and plant tissue softening by maintaining the branched structure of DASP homogalacturonan molecules.
